# Exploring Gut Microbiota Composition and Occurrence of Selected Antimicrobial Resistance Genes in Domestic Pigs and Urban and Sylvatic Wild Boars from Sicily

**DOI:** 10.3390/ani16182938

**Published:** 2026-09-18

**Authors:** Martina Maria Imburgia, Fanny Claire Capri, Rosa Alduina, Roberto Puleio, Rosaria Disclafani, Valeria Blanda, Paola Galluzzo, Sergio Migliore

**Affiliations:** 1 Istituto Zooprofilattico Sperimentale della Sicilia “Mirri”, Via G. Marinuzzi 3, 90129 Palermo, Italy; mmartina.iimburgia@gmail.com (M.M.I.); roberto.puleio@izssicilia.it (R.P.); rosaria.disclafani@izssicilia.it (R.D.); valeria.blanda@izssicilia.it (V.B.); sergio.migliore@izssicilia.it (S.M.); 2 Dipartimento Scienze e Tecnologie Biologiche, Chimiche e Farmaceutiche, Viale delle Scienze, Università di Palermo, 90128 Palermo, Italy; fannyclaire.capri@unipa.it; 3 National Biodiversity Future Center (NBFC), Piazza Marina, 61, 90133 Palermo, Italy; 4 Department of Veterinary Sciences, University of Messina, Viale Giovanni Palatuci 13, 98168 Messina, Italy

**Keywords:** 16 *r*RNA gene sequencing, gut microbiota, antimicrobial resistance, wild boars, One Health, domestic pigs, tetracycline

## Abstract

This exploratory study compared gut microbiota composition and targeted detection of eight antimicrobial resistance genes (ARGs) among three geographically and ecologically distinct Sicilian populations: domestic pigs (DP), urban wild boars (WB-U), and sylvatic wild boars (WB-S). Ileal samples were collected from 36 animals (12 per group) and profiled by *16S rRNA* gene (V3-V4) sequencing and PCR screening for β-lactam (*blaTEM*, *blaCTX-M IV*, *blaOXA*, *blaSHV*) and tetracycline (*tetA*, *tetB*, *tetC*, *tetD*). Firmicutes and Proteobacteria dominated all groups. DP showed high abundance of *Psychrobacter* and *Pseudomonas*, whereas *Lactobacillus* prevailed in WB-U; WB-S showed an intermediate profile. Community composition differed among groups (PERMANOVA, *p* = 0.001). Pairwise comparisons identified differences between DP and each WB group (*p* = 0.0015 for both), but not between WB-U and WB-S (*p* = 0.302). *blaTEM* detection frequencies were 83.3% in WB-U, 16.7% in WB-S, and 50.0% in DP, with a significant WB-U–WB-S difference (*p* = 0.0099). *tetA* was detected in 41.7% of DP and 33.3% of WB-U; *tetC* only in WB-U (25.0%). These findings provide baseline information of differences in gut microbiota composition and in the detection of selected ARGs, while informing future surveillance strategies at the wildlife–livestock–human interface.

## Simple Summary

The gut microbiota is a complex community of microorganisms that plays an important role in digestion, immune function, and health host. At the same time, antimicrobial resistance represents a major One Health concern because resistant bacteria and resistance genes can circulate among humans, animals, and the environment. Wild boars and domestic pigs belong to the same species (*Sus scrofa*) and may occur in different environmental and management settings. The main objective of this exploratory study was to compare the gut microbiota and the occurrence of selected antimicrobial resistance genes among domestic pigs, urban wild boars, and sylvatic wild boars. These animals represented three geographically and ecologically distinct Sicilian populations. Ileal samples collected from wild boars and domestic pigs were analyzed by sequencing the V3–V4 region of the bacterial *16S rRNA* gene. In parallel, selected genes associated with β-lactam and tetracycline resistance were investigated. The study was designed to describe differences among the three sampled populations, rather than to determine the independent or causal effects of domestication, habitat, or anthropogenic exposure. The findings provide baseline information on gut microbial communities and selected antimicrobial resistance genes at the wildlife–livestock–human interface and may inform future surveillance studies.

## 1. Introduction

The mammalian gastrointestinal tract harbors a complex microbial ecosystem that plays a fundamental role in host physiology by contributing to nutrient metabolism, the degradation of complex substrates, vitamin biosynthesis, immune maturation, and mucosal homeostasis [1,2], and has been associated with productive traits such as feed efficiency [3]. Its composition is shaped by host- and environment-related factors, including genetic background, age, diet, health status, antimicrobial exposure, and rearing conditions [4,5,6]. Perturbations of this ecosystem (e.g., disease, weaning stress, or antimicrobial treatment) can lead to dysbiosis and compromise functional stability [5,6]. Recent experimental studies further indicate that gut microbiota modulation can influence inflammatory responses and disease outcomes beyond the intestine, including acute lung injury and mastitis [7,8]. The comparison between domestic pigs (*Sus scrofa domesticus*; DP) and wild boars (*Sus scrofa*; WB) provides an informative framework for describing differences in gut microbiota structure among populations living under different management and environmental conditions [9,10,11]. The two forms are closely related but differ in genetic background, phenotypic traits, diet, living conditions, and exposure to environmental microorganisms and anthropogenic inputs [4,5,6,7,8,9,10]. DP are raised in managed production systems characterized by standardized diets, controlled housing, biosecurity protocols, and veterinary interventions; antimicrobial use and other management practices in these systems may contribute to the selection and maintenance of antimicrobial resistance genes (ARGs) [5,12,13]. In contrast, WB inhabit heterogeneous environments, experience seasonal variation in food availability, and interact with a broader range of environmental microbial reservoirs, providing a substantially different ecological context for microbiota assembly [14,15,16]. In recent decades, WB populations have expanded demographically and geographically across Europe, generating growing ecological, sanitary, and management concerns [17,18]. One consequence has been the emergence of WB in urban and peri-urban areas, where animals may exploit fragmented green spaces, ecological corridors, and anthropogenic food resources, thereby coming into closer contact with humans, domestic animals, waste, and other human-impacted matrices [16,19,20]. In this context, anthropogenic pressure broadly refers to the effects of human activities on wildlife habitats, including urbanization, waste availability, environmental contamination, and human or livestock contact [19,21,22]. In the present study, this term refers to the ecological context of the sampling sites rather than to a quantitatively measured gradient of anthropogenic exposure. Sicily (Italy) is a suitable study area: abundant WB populations are increasingly colonizing diverse ecosystems, from natural habitats to rural, peri-urban, and urban areas [22,23]. The problem is pronounced within protected areas, where the scarcity of natural predators and constraints on population control favor high WB abundance, and consequently, frequent contact with humans and livestock sharing the same territory [23,24]. From a One Health perspective, the increasing presence of wild boars in human-dominated areas has important consequences for ecosystems, animal management, and public health, including a greater risk of pathogen transmission among wildlife, livestock, and humans [24,25,26]. Moreover, screening wild boars for selected ARGs provides descriptive information on the occurrence of these determinants in wildlife and may inform future antimicrobial resistance surveillance [25,27]. In this context, the gut microbiota is not only a functional component of host physiology but also a potential reservoir of ARGs [12,27,28]. Among the ARGs, β-lactam and tetracycline resistance determinants deserve particular attention because they confer resistance to antimicrobial classes widely used in human and veterinary medicine [29,30,31]. Selected *bla* and *tet* genes are frequently investigated in livestock, wildlife, and environmental matrices, making them relevant targets for antimicrobial resistance surveillance within a One Health framework [27,31,32]. Despite growing interest in the suid microbiota and antimicrobial resistance in wildlife [25,27], information on the ileal microbiota of domestic and wild suid populations from different geographical and ecological contexts remains limited [9,10,11]. Because fecal samples, which primarily reflect distal intestinal communities, ileal analysis provides complementary information on an intestinal segment involved in nutrient absorption and mucosal immune interactions [33,34]. In this context, the present exploratory study compared ileal microbiota composition and detected a selected set of eight ARGs in three Sicilian suid populations: domestic pigs (DP), urban wild boars (WB-U), and sylvatic wild boars (WB-S). The aim was to describe differences among the sampled populations and provide baseline information on ileal microbiota composition and the detection of the selected ARGs.

## 2. Materials and Methods

### 2.1. Sample Collection

**Figure 1:**
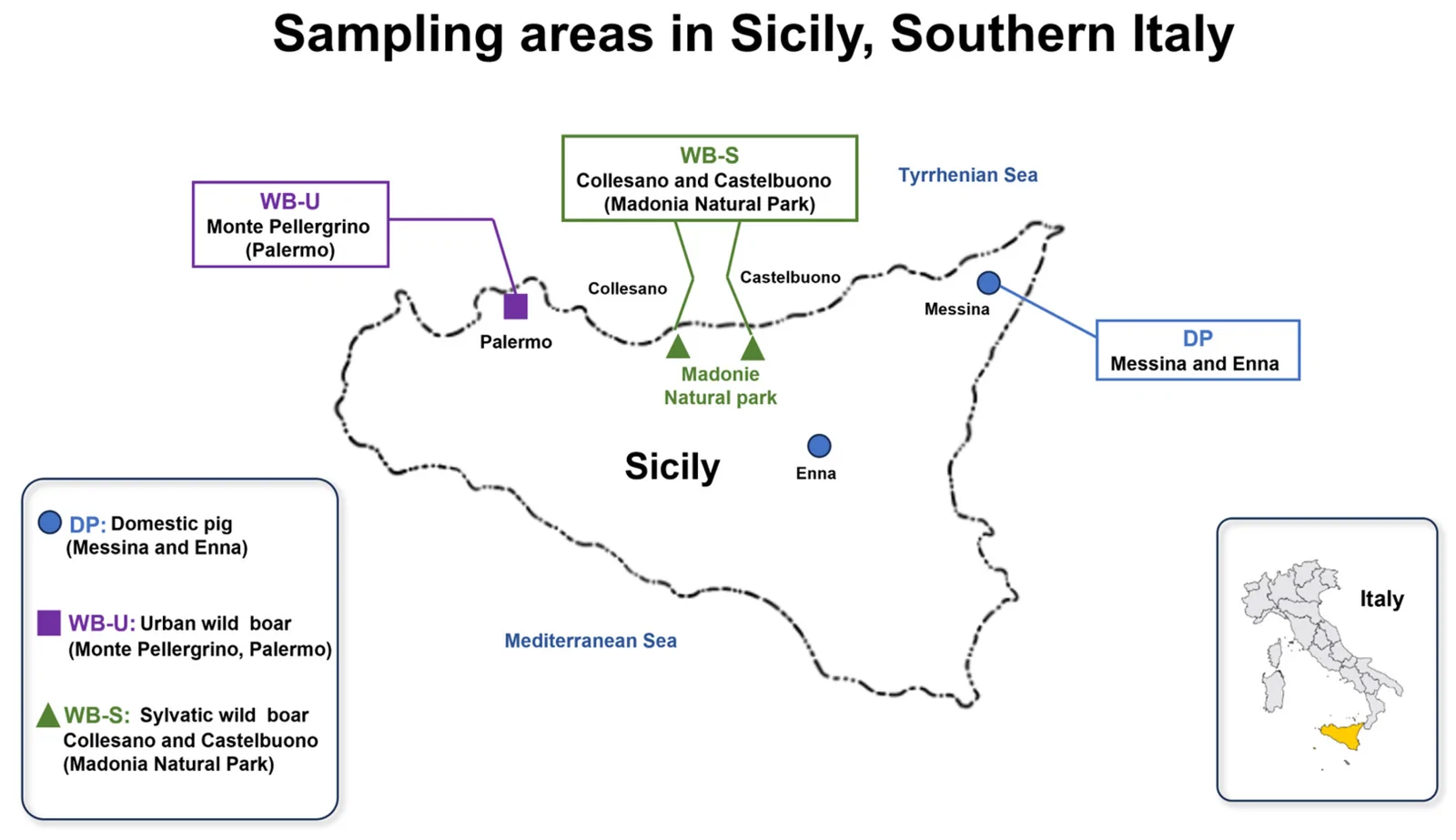
Geographical distribution of the sampling areas in Sicily, Southern Italy. Blue circles identify the sampling areas of domestic pigs (DP: Messina and Enna; *n* = 12 in total), the purple square identifies the sampling area of urban wild boars (WB-U: Monte Pellegrino, Palermo; *n* = 12), and green triangles identify the sampling areas of sylvatic wild boars (WB-S: Collesano and Castelbuono, within the Madonie Natural Park; *n* = 12 in total). The symbols indicate sampling areas rather than individual animals, and their colors and shapes were used solely to distinguish the three groups visually. The inset shows the location of Sicily within Italy.

Intestinal tissue (~15 cm in length) was collected post-mortem from the distal ileum of each animal (WB and DP subjects) and immediately stored at −80 °C until analysis. The sampling design included three geographically and ecologically distinct Sicilian suid populations: (i) domestic (DP), (ii) urban (WB-U), and (iii) sylvatic (WB-S). For each population, 12 individuals were included (*n* = 12/group), for a total of 36 samples, collected during the 2022–2023 biennium (November–March) (Table S1). The DP group samples (1DP–12DP) were collected from the Nero dei Nebrodi pigs reared under extensive farming systems in the provinces of Messina (38°11′37″ N 15°33′15″ E) and Enna (37°35′ N 14°26′ E) (Sicily, Southern Italy). The Nero dei Nebrodi is an indigenous Sicilian pig breed, with origins dating back to the Greek and Carthaginian periods, traditionally reared under extensive free-range systems in the mountainous Nebrodi area of northeastern Sicily [35]. The WB-U samples (1WB-U–12 WB-U) were collected exclusively in the Monte Pellegrino located in Palermo (38°10′12″ N 13°21′05″ E; Sicily, Southern Italy). Monte Pellegrino is a limestone promontory overlooking the Palermo metropolitan area, where natural habitats occur in close proximity to urban, industrial, and coastal settings [22]. The WB-S samples (1 WB-S -12 WB-S) were collected in Collesano (37°55′ N 13°56′ E) and Castelbuono (37°56′ N 14°06′ E) in Madonie Natural Park (Palermo, Southern Italy) (Figure 1). Madonie Natural Park is a protected area characterized predominantly by natural and semi-natural landscapes [36]. The terms “urban” and “sylvatic” refer exclusively to the geographical and ecological contexts of the sampling sites and not to quantitative measurements of anthropogenic exposure. All sampled wild individuals (urban and sylvatic groups) were obtained from animals culled as part of the wild suid population control programs implemented in the Monte Pellegrino area (approved with D.D.G. n.812 of 1 August 2017, with D.D.G. n. 1361 of 29 August 2019 and with D.D.G. n.1154 of 21 July 2021 until 22 June 2023) and Madonie Natural Park (approved with resolution of the President of the Regional Council n. 6 of 15 February 2016). In contrast, DP was sampled during regular slaughter at the slaughterhouse of Enna district, an officially authorized slaughterhouse operating under the supervision of the competent health authorities in Sicily. Animal age was approximately 12–24 months. Sex information was incomplete and not consistently available; therefore, sex and age were not included as covariates in between population comparisons. No direct dietary data or quantitative indicators of anthropogenic exposure were collected for any of the three populations; these variables were therefore not included in the analyses.

### 2.2. Genomic DNA Extraction, PCR Amplification, and Sequencing

Metagenomic DNA was extracted from the collected ileal samples using the QIAamp PowerFecal Pro DNA Kit (QIAGEN, Hilden, Germany), following the manufacturer’s instructions. An extraction blank containing all kit reagents but no biological material was processed alongside the ileal samples, and a no-template control was included during PCR amplification. Neither control produced a detectable amplicon by agarose gel electrophoresis and, consequently, they were not included in the sequencing library. Metagenomic DNA concentration was measured with a NanoDrop 2000c spectrophotometer (Thermo Fisher Scientific, Waltham, MA, USA) and used as template for amplification of the *16S rRNA* gene V3–V4 hypervariable region (464 bp) using universal primers 341F (5′-TCGTCGGCAGCGTCAGATGTGTATAAGAGACAGCCTACGGGNBGCASCAG-3′) and 805R (5′-GTCTCGTGGGCTCGGAGATGTGTATAAGAGACAGGACTACNVGGGTATCTAATCC-3′) [37]. A control was included in the PCR amplification to monitor for potential contamination.

Amplicon presence was confirmed by electrophoresis at 1.5% (*w*/*v*) agarose gel. Neither the extraction blank nor the no-template control produced a detectable amplicon. Amplicon products were sequenced by next-generation sequencing (NGS) on an Illumina MiSeq platform (BMR Genomics s.r.l., Padova, Italy).

### 2.3. Raw-Data Processing and Microbiota Analysis

The raw *16S rRNA* data were processed using the QIIME2 (2025.10) suite as paired-end sequences [38]. Demultiplexed reads were imported into QIIME 2 and their quality profiles were visually inspected using the qiime demux summarize command. Primer and adapter sequences were removed using cutadapt. Forward and reverse reads were then denoised using the DADA2 plugin (qiime dada2 denoise-paired) [39]. Reads exceeding the expected error threshold were discarded. DADA2 was used to perform quality filtering, dereplication, paired-end read merging, chimera detection and removal, and inference of exact Amplicon Sequence Variants (ASVs). ASVs were taxonomically assigned using the feature-classifier plugin against the SILVA 138.2 reference database (trained for V3–V4) using a 99% sequence-similarity threshold. ASVs that could not be assigned at the selected taxonomic rank were retained and reported as “Unclassified”. QIIME2 was used to generate rarefaction curves, Good’s coverage index, and α-diversity metrics. Rarefaction analysis was performed by plotting the number of observed ASVs against the number of filtered reads for each sample. Based on the rarefaction curves, α-diversity metrics (i.e., ACE, Chao1, Shannon entropy, and Simpson index) were calculated on a rarefied frequency-feature table (Table S2). Principal Coordinate Analysis (PCoA) was performed in the software Emperor (v1.18-408), starting from the previously transformed Bray–Curtis distance matrix using square-root transformation. We also evaluated homogeneity of multivariate dispersion among groups using PERMDISP with the same Bray–Curtis distance matrix and 999 permutations. PERMANOVA results were interpreted together with the PERMDISP analysis because differences in within-group dispersion may contribute to significant PERMANOVA results. The sequence dataset was deposited in the GenBank database (BioProject: PRJNA1505544).

### 2.4. Detection of Antimicrobial Resistance Genes

The presence of selected ARGs associated with β-lactam and tetracycline resistance was investigated. Specifically, β-lactam resistance genes were detected at family level by quadruplex PCR (SET1: *blaCTX-M IV*, *blaTEM*, *blaOXA*, *blaSHV*), as previously described [40]. In addition, PCR screening was performed for tetracycline resistance genes (*tetA*, *tetC*, *tetD*, *tetB*) as previously described [41]. For the analysis of the ARGs, DreamTaq DNA polymerase (Thermo Fisher Scientific) was used according to the manufacturer’s instructions. Primer pairs are listed in Table 1. The thermal cycling program consisted of an initial denaturation step at 95 °C for 2 min, followed by 35 cycles of denaturation at 95 °C for 30 s, primer annealing at the temperature reported in Table 1 for 30 s, and extension at 72 °C for 30 s, with a final extension step at 72 °C for 5 min. Positive and negative controls were added in each amplification. A no-template control containing all PCR reagents but no DNA template was included in each PCR run. PCR products were visualized by agarose gel electrophoresis using a 1.5% (*w*/*v*) agarose gel. No amplification was observed in the negative controls.

### 2.5. Statistical Analysis

For each alpha-diversity index, differences among the three populations were evaluated using one-way analysis of variance (ANOVA). The suitability of the parametric model was assessed by examining the distribution of residuals using the Shapiro–Wilk test, while homogeneity of variances was evaluated using the Brown–Forsythe test. The presence of influential extreme outliers was also assessed by inspection of residual plots. Because the study design was balanced, with 12 independent animals in each group, one-way ANOVA was retained when no substantial violation of its assumptions was detected. All reported pairwise *p*-values were adjusted for multiple comparisons, and statistical significance was defined as an adjusted *p*-value < 0.05. Differences in overall microbial community composition among the three groups were evaluated by permutational multivariate analysis of variance (PERMANOVA) using the Bray–Curtis dissimilarity matrix. The analysis was performed in QIIME 2 using the beta-group-significance function with 999 unrestricted permutations of group labels. An overall PERMANOVA was followed by pairwise comparisons among DP, WB-U, and WB-S. Pairwise *p*-values were adjusted for multiple comparisons using the Benjamini–Hochberg false-discovery-rate procedure implemented in QIIME 2.The proportion of variation explained by group membership was expressed as R^2^ and derived from the PERMANOVA pseudo-F statistic according to the following equation: R^2^ = 1/[1 + (n − g)/((g − 1)F)], where n is the number of samples, g is the number of groups, and F is the pseudo-F statistic. For each targeted ARG, detection frequency was calculated as the number of animals in which the gene was detected divided by the total number of animals examined in each group. Exact 95% confidence intervals for detection frequencies were calculated using the Clopper–Pearson method. Detection frequencies were compared pairwise among the three sampled groups using two-sided Fisher’s exact tests. The three pairwise comparisons were DP versus WB-S, DP versus WB-U, and WB-S versus WB-U. For each gene, the resulting *p*-values were adjusted for the three pairwise comparisons using the Holm–Šídák method. Statistical significance was set at an adjusted *p*-value < 0.05. Given the limited number of animals in each group, these analyses were considered exploratory. All statistical analyses were performed using GraphPad Prism version 8.4.2 (GraphPad Software, San Diego, CA, USA).

## 3. Results

### 3.1. Microbial Diversity

**Figure 2:**
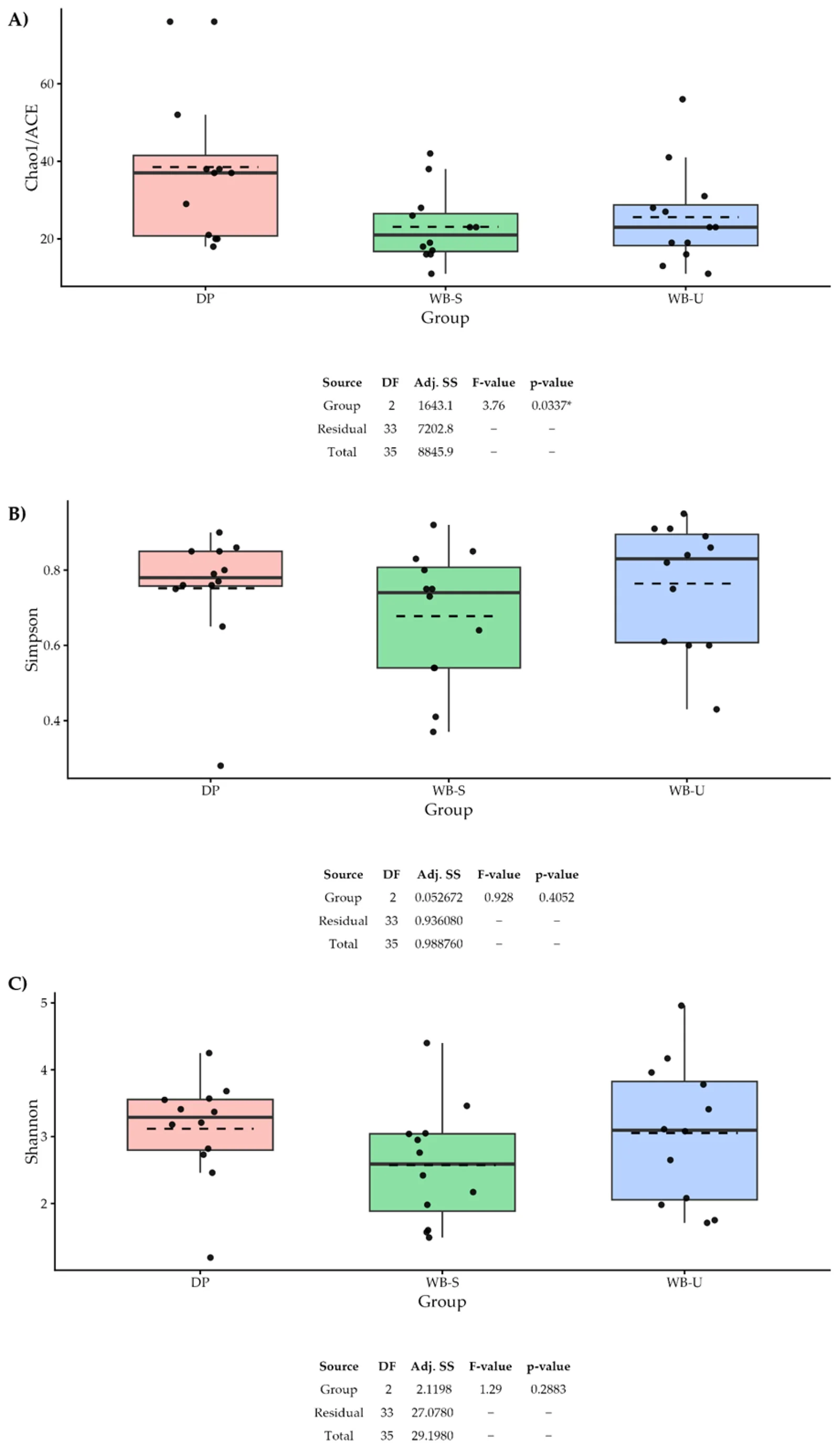
Boxplots show the richness estimates, including Chao1 and ACE, which yielded identical values for all samples in the present dataset (**A**), Simpson index (**B**), and Shannon index (**C**) in domestic pigs (DP), sylvatic wild boars (WB-S), and urban wild boars (WB-U). Each point represents one individual animal (*n* = 12 per group); boxes indicate the interquartile range, solid horizontal lines represent the median, and dashed lines represent the mean. Overall differences were evaluated using one-way ANOVA followed, when significant, by Tukey’s multiple-comparisons test. DF: total degrees of freedom; Adj. SS: Adjusted sums of squares; An asterisk indicates statistical significance at *p* ≤ 0.05.

**Figure 3:**
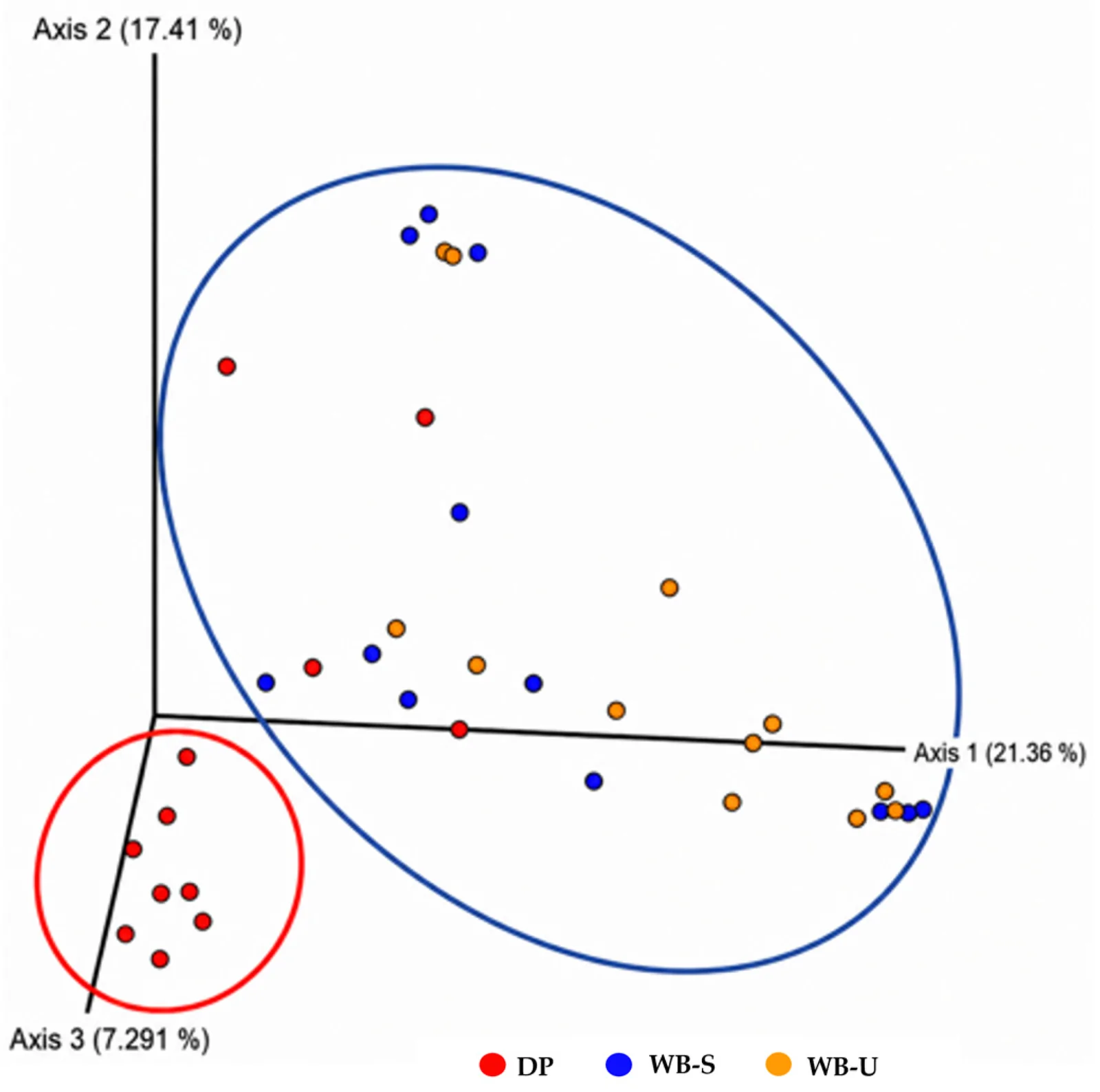
PCoA based on the Bray–Curtis distance matrix among domestic pigs (DP), sylvatic wild boars (WB-S), and urban wild boars (WB-U); each point represents one animal. Axes 1, 2, and 3 explain 21.36%, 17.41%, and 7.29% of the total variation, respectively. The red and blue outlines are descriptive visual guides that highlight the main areas occupied by DP and the two broadly overlapping wild-boar groups, respectively; they do not represent confidence ellipses or statistically defined clusters. WB-S and WB-U were analyzed separately and are displayed in different colors.

Alpha- and Beta- diversity were assessed in all 36 samples. Observed ASV richness was higher in DP (38.50 ± 20.23) than in WB-U (25.58 ± 12.66) and WB-S (23.08 ± 9.24); ACE and Chao1 estimates were essentially identical to observed richness in all groups (Table S2), and the differences among groups were significant (*p* = 0.0337, Figure 2A). Shannon diversity was comparable in DP and WB-U (3.12 ± 0.77 and 3.05 ± 1.05, respectively) and lower in WB-S (2.57 ± 0.87), while Simpson values were similar across groups (DP: 0.75 ± 0.16; WB-U: 0.76 ± 0.17; WB-S: 0.68 ± 0.18); neither index differed significantly among groups (Shannon: *p* = 0.288; Simpson: *p* = 0.405; Table S2; Figure 2A–C). Observed ASV richness differed significantly among the three groups (one-way ANOVA: F_2_,_33_ = 3.76, *p* = 0.0337). Tukey’s post hoc test showed significantly higher richness in DP than in WB-S (mean difference = 15.42 ASVs, *p* = 0.0397). In contrast, the difference between DP and WB-U did not reach statistical significance (mean difference = 12.92 ASVs, *p* = 0.0968), and no significant difference was detected between WB-S and WB-U (mean difference = −2.50 ASVs, *p* = 0.9099). Principal coordinate analysis (PCoA) based on Bray–Curtis dissimilarities separated the three suid groups (Figure 3). DP samples formed a distinct cluster, whereas WB-S and WB-U samples showed broader dispersion and partial overlap. Bray–Curtis dissimilarities differed significantly among the three sampled groups (PERMANOVA: pseudo-F_2_,_33_ = 3.294, R^2^ = 0.166, *p* = 0.001), with group membership explaining approximately 16.6% of the variation in ileal microbial community composition. Pairwise analyses showed significant differences between DP and WB-S (pseudo-F_1_,_22_ = 3.563, R^2^ = 0.139, *p* = 0.001, q = 0.0015) and between DP and WB-U (pseudo-F_1_,_22_ = 5.596, R^2^ = 0.203, *p* = 0.001, q = 0.0015). No significant difference was detected between WB-S and WB-U (pseudo-F_1_,_22_ = 1.139, R^2^ = 0.049, *p* = 0.302, q = 0.302). Overall, these results indicate that the main shift in community composition was associated with the DP population, while WB-S and WB-U samples retained partially overlapping community profiles.

### 3.2. Microbial Composition

**Figure 4:**
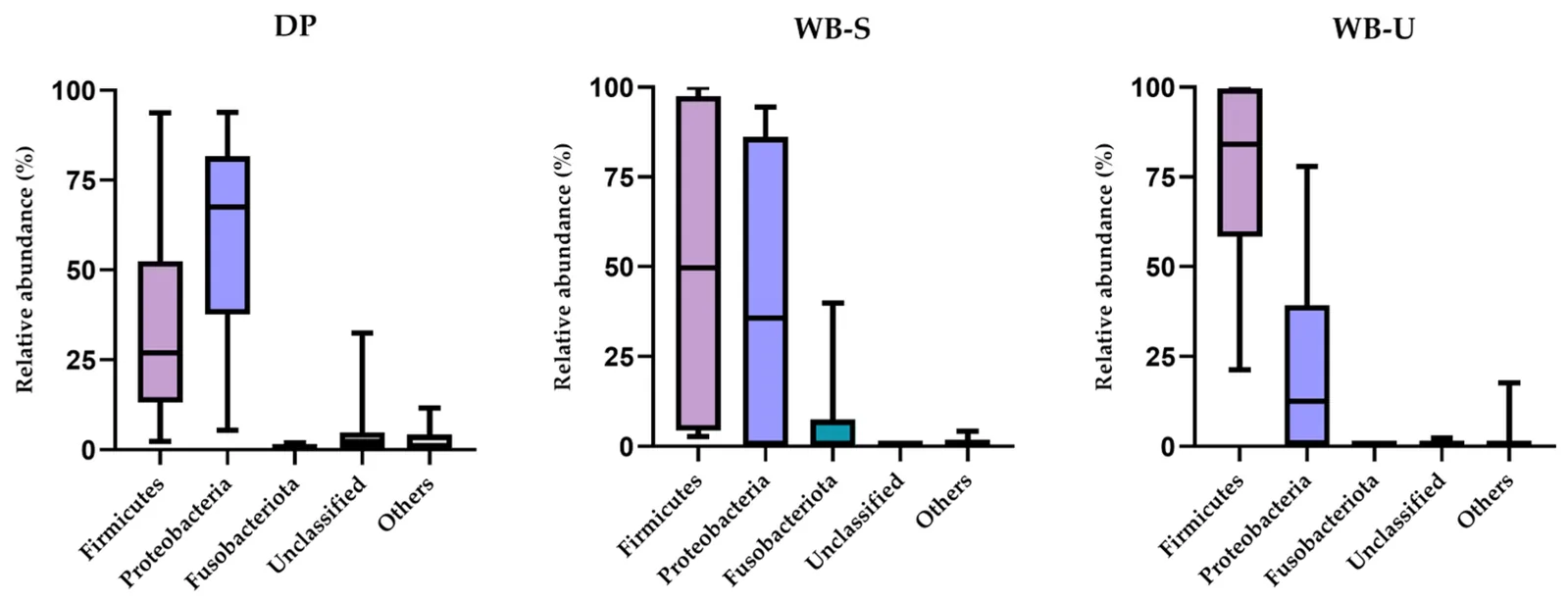
Relative abundance (%) of the bacterial phyla in the gut microbiota of DP, WB-S, and WB-U from Sicily (*n* = 12 per group). Boxes show the interquartile range, the line the median, and whiskers the minimum and maximum. “Unclassified” denotes sequences that could not be assigned at the phylum level; “Others” pools the remaining low-abundance phyla. The observed differences among groups are descriptive, as no formal statistical comparisons were performed at the phylum level.

**Figure 5:**
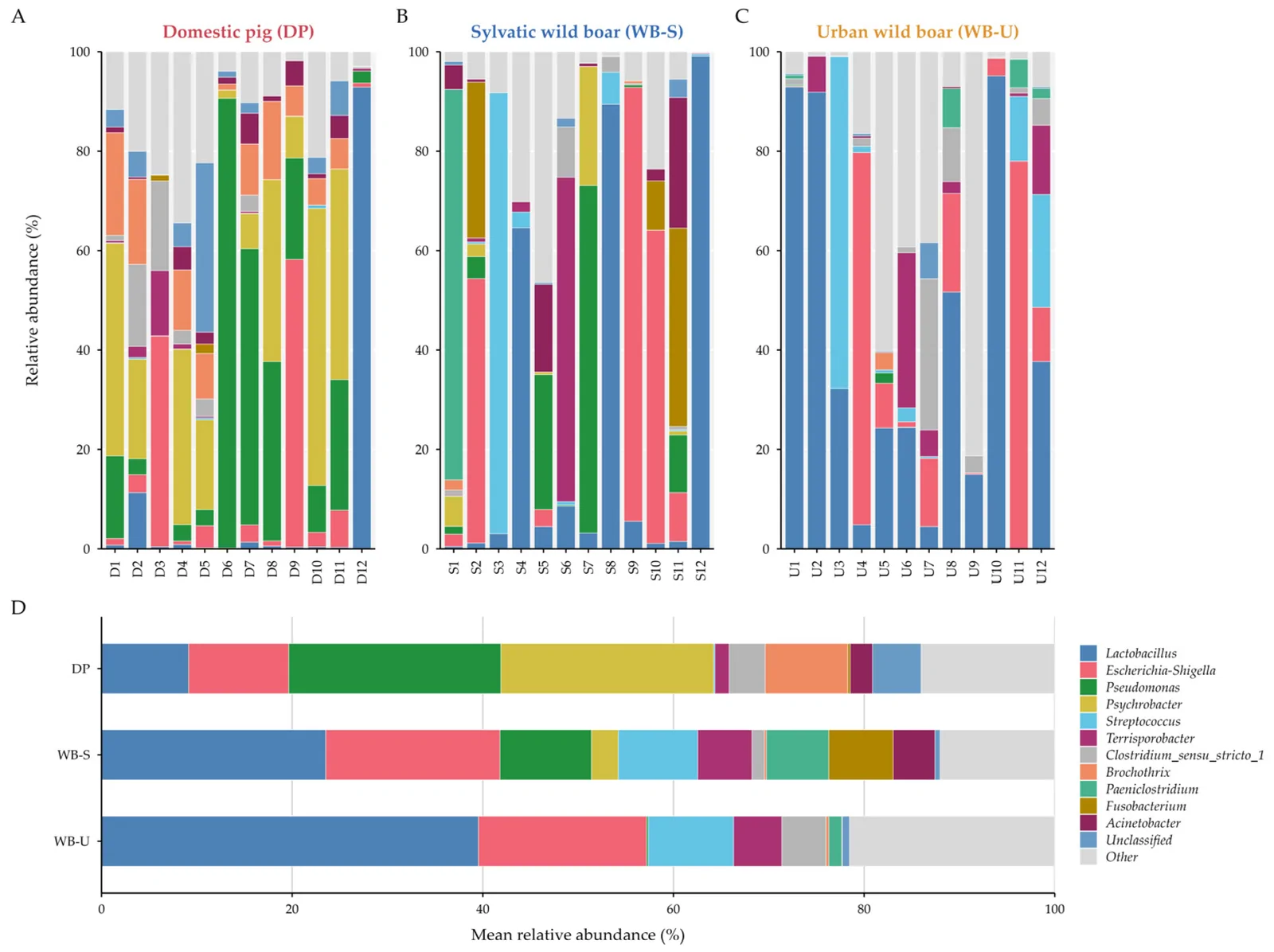
Genus-level composition of the gut microbiota of DP (**A**), WB-S (**B**) and WB-U (**C**) from Sicily (*n* = 12 per group). (**D**) Mean relative abundance of the same taxa in each group.

Taxonomic composition was compared among the three groups at the phylum and genus levels (Figure 4 and Figure 5). Firmicutes and Proteobacteria together accounted for more than 90% of the reads in all groups, but their proportions differed. DP were dominated by Proteobacteria (60.09% ± 27.14), with Firmicutes second (33.22% ± 25.63), whereas the opposite was observed in WB-U (Firmicutes 75.29% ± 28.98; Proteobacteria 22.84% ± 28.87); WB-S showed an intermediate profile (Firmicutes 51.42% ± 46.46; Proteobacteria 40.89% ± 41.41) (Figure 4). Fusobacteriota was the only other phylum to reach an appreciable abundance, and did so exclusively in WB-S, where it was detected in 3 of 12 animals and reached 39.9% in one individual; it was never detected in WB-U. The remaining phyla (i.e., Campylobacterota, Bacteroidota, Cyanobacteria, Actinobacteriota, Verrucomicrobiota, Euryarchaeota, and Spirochaetota) accounted for less than 5% of the sequences in all animals except one WB-U (17.6%). Among these, Campylobacterota was the most consistently detected, being present in 7 of 12 DP but in only one animal of each WB group. Sequences that could not be assigned at the phylum level represented a minor fraction overall but were more frequent in DP (median 2.3%, up to 32.3%) than in either wild group.

At the genus level, the most abundant genera were *Lactobacillus* (24.0%), *Escherichia*-*Shigella* (15.5%), *Pseudomonas* (10.7%), *Psychrobacter* (8.4%), and *Streptococcus* (5.8%), followed by *Terrisporobacter* (4.1%), *Clostridium sensu stricto* 1 (3.2%), and *Brochothrix* (3.1%); unassigned sequences accounted for a further 2.1%. Their distribution, however, differed among the three groups (Figure 5).

**Figure 6:**
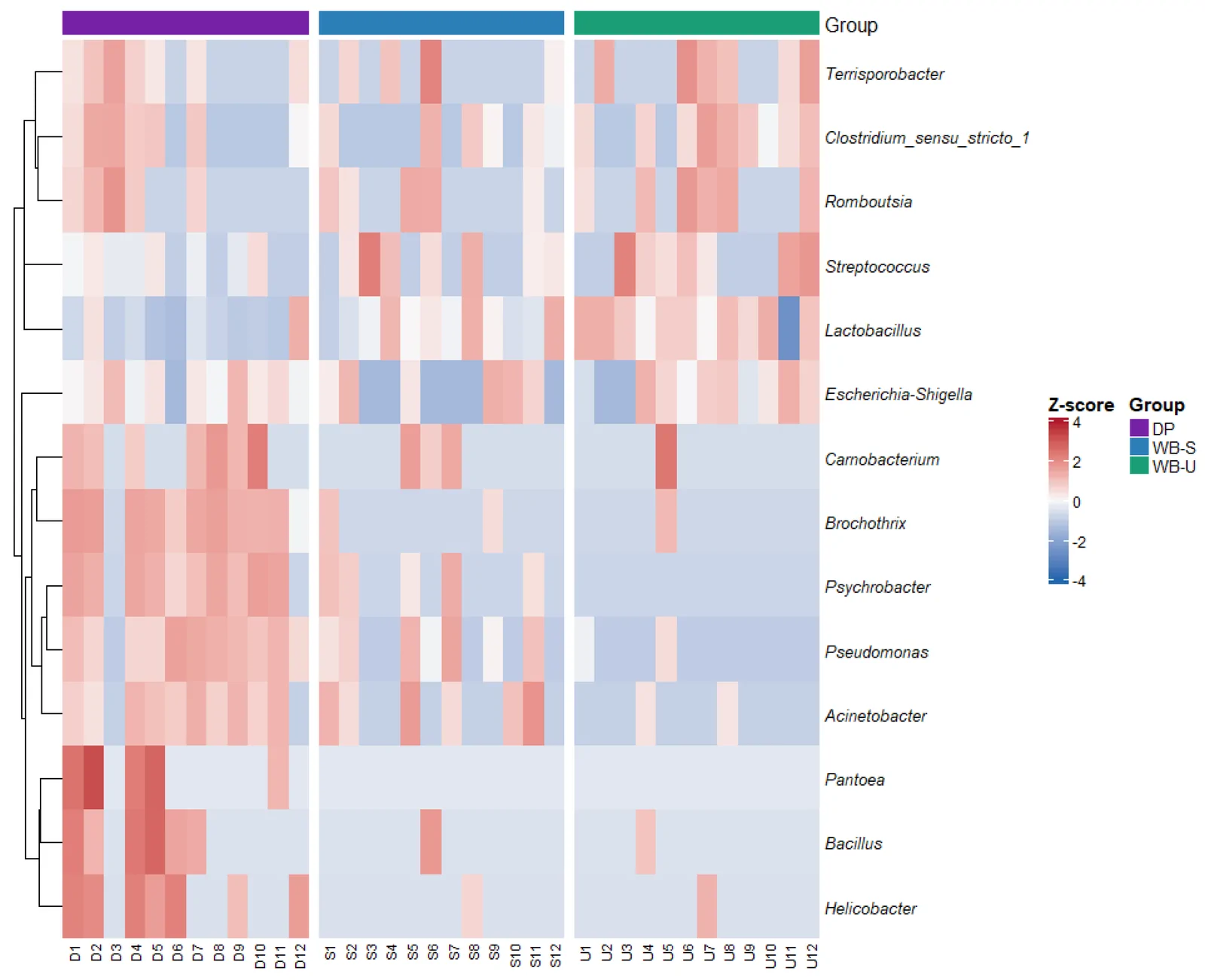
Heatmap of genus-level relative-abundance patterns across the 36 individual ileal samples from domestic pigs (DP), sylvatic wild boars (WB-S), and urban wild boars (WB-U) (*n* = 12 per group). Columns represent individual animals and rows represent bacterial genera. Relative-abundance values were standardized separately for each genus across all samples using row-wise Z-score transformation. Red indicates an abundance above the across-sample mean for the corresponding genus, white indicates a value close to the mean, and blue indicates an abundance below the mean. Genera were hierarchically clustered using Euclidean distance and complete-linkage clustering, as shown by the left dendrogram. Samples were not clustered but were arranged by group; the colored top annotation identifies DP, WB-S, and WB-U.

To summarize genus-level patterns descriptively, genera detected in at least half of the animals within a group (≥6/12) and accounting for a mean relative abundance of at least 1% within that group were retained for visualization and descriptive comparison. This operational criterion was not a differential-abundance test and should not be interpreted as evidence of a statistical association between individual genera and group membership (Figure 6).

Only three genera were shared by all three groups (i.e., *Lactobacillus*, *Escherichia*-*Shigella*, and *Clostridium sensu stricto* 1). In DP, *Psychrobacter* (22.31% ± 19.49; 10/12 animals) and *Pseudomonas* (22.25% ± 27.21; 11/12) were found, followed by *Escherichia*-*Shigella* (10.51% ± 18.92; 11/12), *Lactobacillus* (9.13% ± 26.55; 12/12), *Brochothrix* (8.66% ± 6.79; 11/12), *Clostridium sensu stricto* 1 (3.79% ± 6.45; 7/12), *Acinetobacter* (2.34% ± 2.20; 10/12), *Carnobacterium* (2.14% ± 4.69; 7/12), *Terrisporobacter* (1.49% ± 3.71; 7/12), and *Bacillus* (1.25% ± 2.25; 6/12). In addition, six genera were restricted to DP, being detected in at least five DP but in no more than one WB of either group: *Candidatus Bacilloplasma* (2.98%; 5/12), *Bacillus*, *Pantoea* (1.22%; 5/12), *Helicobacter* (0.96%; 7/12), *Serratia* (0.24%; 8/12), and *Mycoplasma* (0.05%; 5/12). In WB-U, *Lactobacillus* reached the highest mean abundance recorded in the study (39.53% ± 35.60; 11/12), together with *Escherichia*-*Shigella* (17.62% ± 28.22; 10/12), *Streptococcus* (8.96% ± 19.68; 7/12), *Terrisporobacter* (5.06% ± 10.51; 6/12), *Clostridium sensu stricto* 1 (4.63% ± 8.71; 9/12), and *Romboutsia* (1.44% ± 2.13; 6/12). *Psychrobacter* was never detected in this group, and *Pseudomonas* was found in only two animals. WB-S occupied an intermediate position and shared members with both other groups: *Lactobacillus* (23.39% ± 37.27; 12/12) and *Escherichia*-*Shigella* (18.28% ± 32.06; 6/12) as in WB-U, *Streptococcus* (8.33% ± 25.53; 7/12) and *Clostridium sensu stricto* 1 (1.30% ± 2.32; 6/12) likewise, but also *Pseudomonas* (9.60% ± 20.60; 7/12) and *Acinetobacter* (4.37% ± 8.55; 6/12) as in DP. Notably, *Lactobacillus* was the only genus detected in every sylvatic animal, and the group core was the smallest of the three. Overall, the three sampled populations showed different descriptive taxonomic profiles, with DP characterized mainly by *Psychrobacter* and *Pseudomonas*, WB-U by *Lactobacillus*, and WB-S showing an intermediate and more heterogeneous composition.

### 3.3. Antimicrobial Resistance Genes

Within the selected ARGs, molecular screening detected only three of the selected ARGs. Among the β-lactamase genes investigated, *blaTEM* was the only gene detected. Its detection frequency was highest in WB-U (10/12, 83.33%; 95% CI: 51.59–97.91%), followed by DP (6/12, 50.00%; 95% CI: 21.09–78.91%) and WB-S (2/12, 16.67%; 95% CI: 2.09–48.41%). Pairwise comparisons showed a significant difference in *blaTEM* detection frequency between WB-S and WB-U (two-sided Fisher’s exact test, Holm–Šídák-adjusted *p* = 0.0099) (Table 2). The comparisons between DP and WB-S and between DP and WB-U did not reach statistical significance (adjusted *p* = 0.3488 for both comparisons). The remaining β-lactamase genes investigated, *blaOXA*, *blaSHV*, and *blaCTX-M IV*, were not detected in any sample (Table 2). Among the tetracycline resistance genes, *tetA* was detected in DP (5/12, 41.67%; 95% CI: 15.17–72.33%) and WB-U (4/12, 33.33%; 95% CI: 9.92–65.11%) but not in WB-S (0/12, 0%; 95% CI: 0–26.46%). None of the pairwise comparisons reached statistical significance after Holm–Šídák correction (DP versus WB-S: adjusted *p* = 0.1078; DP versus WB-U: adjusted *p* = 1.0000; WB-S versus WB-U: adjusted *p* = 0.1777). The *tetC* gene was detected only in WB-U (3/12, 25.00%; 95% CI: 5.49–57.19%) and was not detected in DP or WB-S (0/12, 0%; 95% CI: 0–26.46% for each group). No pairwise comparison of *tetC* detection frequencies reached statistical significance (DP versus WB-S: adjusted *p* = 1.0000; DP versus WB-U and WB-S versus WB-U: adjusted *p* = 0.5207 for both comparisons). Neither *tetB* nor *tetD* was detected in any group (Table 2). Detection results for all investigated antimicrobial resistance genes are reported for each ileal sample in Supplementary Table S3. Overall, *blaTEM* was the most frequently detected gene and the only investigated gene showing a significant pairwise difference in detection frequency. However, given the small sample size, the low number of positive animals for several genes, and the wide confidence intervals, the non-significant comparisons should not be interpreted as evidence of equivalence between the sampled populations.

## 4. Discussion

This exploratory observational study compared the gut microbiota composition and the detection of selected β-lactam and tetracycline resistance genes among three geographically and ecologically distinct Sicilian suid populations comprising domestic pigs (DP), urban wild boars (WB-U), and sylvatic wild boars (WB-S). Richness estimators (ASVs, Chao1 and ACE) were higher in DP than in either WB group, whereas Shannon and Simpson indexes did not differ, indicating that DP harbored a greater number of taxa without a correspondingly more even community. Comparable discrepancies between richness and evenness metrics have been reported in earlier comparisons of DP and WB [11], and higher richness in managed animals has been discussed in relation to potential differences in diet, husbandry practices, and environmental exposure [9,11]. The β-diversity analysis revealed differences in the overall composition of the microbial communities among the groups. In the PCoA based on Bray–Curtis distances, DP formed a compact cluster, while WB-S and WB-U overlapped extensively, so that the principal axis of variation separated the domestic from the wild form rather than the two wild habitats from one another. Similar differences in gut microbial community structure have been reported in previous comparisons involving domestic pigs and wild boars living under different feeding, management, and environmental conditions [9,10,11]. DP resembled one another closely, whereas wild boars of both groups differed markedly among individuals. However, because individual dietary data were not collected, this variability cannot be attributed to differences in feeding patterns. Because the populations also differed in geographical origin, diet, and management, the observed separation cannot be attributed to any single factor. At the phylum level, DP was dominated by Proteobacteria, WB-U by Firmicutes, and WB-S was intermediate and highly variable. The scarcity of Bacteroidota, below 1% in all groups, is expected in the ileum compartment, since the members of this phylum are primarily fibrolytic fermenters of the large intestine, and segment-resolved studies of the porcine gastrointestinal tract consistently report Firmicutes and Proteobacteria as the dominant phyla of gut digesta, with Bacteroidota becoming dominant only from the caecum onwards [1,34,42]. Among the minor phyla, Campylobacterota was the only one to show a consistent group-related pattern, being detected in 7 of 12 DP but in a single animal of each WB group; this signal corresponds entirely to *Helicobacter*, a genus whose members are frequently reported in the gastric mucosa of farmed pigs [43,44]. Therefore, their detection in ileal samples may reflect transit from the stomach rather than stable colonization of the ileum, although their anatomical origin cannot be determined from the present analysis. However, no information on the health status of the sampled DP animals was available; therefore, the higher detection frequency observed in this population cannot be interpreted as evidence of infection or disease. Fusobacteriota were more strongly represented in WB-S than elsewhere, although they were detected in only three animals and the difference was not statistically supported. Fusobacteria are abundant in the gut microbiota of specialized carrion feeders such as vultures [45], and wild boars are known to interact with carcasses [46]. The presence of free-ranging cattle and sheep in the Madonie Park [47] and the expansion of wild ungulate populations in the same area [48,49] provide a plausible ecological context for this exploratory observation, suggesting that the greater representation of Fusobacteriota in WB-S may be compatible with occasional carcass-related exposure, although this interpretation remains speculative. The Proteobacteria-dominated profile of DP was driven principally by *Psychrobacter* and *Pseudomonas*, genera that were poorly represented in wild boars. Both genera are environmentally widespread and have been reported in soil, water, and plant-associated habitats [50,51,52]. Given the extensive or semi-free-range conditions in which the DP were raised, one possible exploratory interpretation could be that the greater representation of these genera, together with that of *Bacillus*, *Pantoea*, and *Serratia*, may reflect greater contact with soil and other environmental matrices [10]. This hypothesis is consistent with previous studies reporting associations between exposure to agricultural soil, the complexity of the rearing environment, and variation in the suid gut microbiota [10,53]. The high abundance of Proteobacteria in DP should not be automatically interpreted as evidence of dysbiosis. Although expansion of this phylum has been proposed as a possible marker of intestinal instability in several host systems [54,55], no clinical, inflammatory, histological, or metabolomic parameter was assessed here that would support such an interpretation. The predominance of Firmicutes in WB-U was associated with the high abundance of *Lactobacillus*, which reached approximately 40% of the community in this group against 23% in WB-S and 9% in DP. Several members of this genus use readily fermentable carbohydrates and may be favored by the availability of dietary energy substrates [56]. WB inhabiting urban and peri-urban areas have access to both natural resources and anthropogenic food, including organic waste and food scraps rich in fermentable carbohydrates [16,21,57]. In this context, one possible exploratory interpretation is that the greater representation of *Lactobacillus* in WB-U may reflect a diet strongly influenced by human-derived resources [16,21,56]. The remaining representative taxa of this group (i.e., *Streptococcus*, *Clostridium sensu stricto 1*, *Terrisporobacter*, and *Romboutsia*) are all typical members of the suid gut community [42,58], so that the WB-U profile can be described as a conventional small-intestinal community in which the lactobacilli are unusually dominant. Conversely, the environmental genera characteristic of DP were almost entirely absent from this group: *Psychrobacter* was never detected, and *Pseudomonas* was found in only two animals. WB-S occupied an intermediate position, sharing *Lactobacillus* and *Streptococcus* with WB-U and *Pseudomonas* and *Acinetobacter* with DP, and showed the smallest and least consistent core of the three groups; *Lactobacillus* was the only genus detected in every sylvatic animal. This heterogeneity observed in WB-S may reflect the diversity of food resources and environmental exposures available in natural habitats [14,15,16], together with the episodic feeding patterns of free-ranging animals which could contribute to interindividual variation in a rapid transit compartment such as the ileum [14,42,59]. Nevertheless, the high relative abundance of *Psychrobacter* and *Pseudomonas* observed in DP should be interpreted cautiously. Although it may reflect genuine environmental exposure, alternative methodological explanations, including differences in carcass handling, post-mortem interval, sample contamination [60,61], or reagent-associated contamination, cannot be excluded in the absence of dedicated contamination controls. The *Escherichia*-*Shigella* group includes both commensal and potentially pathogenic lineages of public-health relevance [62] and was among the three most abundant taxa across all three sampled populations. It was detected in comparable proportions in DP, WB-U, and WB-S, suggesting that it may represent a common component of the ileal microbiota of the sampled suids. However, genus-level assignment based on the V3–V4 region of the *16S rRNA* gene does not allow commensal and potentially pathogenic lineages to be distinguished [63]. Therefore, the occurrence and relative abundance of this group do not demonstrate that the sampled animals act as reservoirs of zoonotic Enterobacteriaceae and cannot be interpreted as evidence of infection or disease, particularly because information on their health status was unavailable. Moreover, the relatively high abundance of environmentally widespread genera, particularly *Psychrobacter*, *Pseudomonas*, *Acinetobacter*, *Bacillus*, *Pantoea*, and *Serratia*, should be interpreted cautiously and cannot be attributed exclusively to biological differences among the sampled populations.

Among the four β-lactamase genes screened, only *blaTEM* was detected. Its detection frequency was highest in WB-U (83.3%), followed by DP (50.0%), and WB-S (16.7%). This pattern is consistent with previous Italian reports of *blaTEM* in WB [64,65]. The higher detection frequency in urban animals may be consistent with greater exposure to urban environments and anthropogenic waste. These have been proposed as potential sources of β-lactam resistance determinants in WB populations [64]. The intermediate detection frequency in DP may instead reflect management-related and environmental exposures associated with rural farming systems [10,66]. The absence of *bla*CTX-M, *bla*OXA, and *bla*SHV indicates limited diversity among the β-lactam determinants detectable by the targeted screening applied here. The absence of *bla*CTX-M in particular contrasts with Italian studies of *Escherichia coli* isolated from WB, in which this gene was frequently reported [64,65,67]. This discrepancy may reflect geographical variation in gene circulation, the limited sample size, or methodological differences, since the present study screened total ileum DNA rather than individual isolates. Among tetracycline determinants, *tet*A showed detection frequencies of 41.7% in DP and 33.3% in WB-U and was not detected in sylvatic animals. This distribution may be consistent with the frequent occurrence of this gene in livestock and anthropogenically influenced environments, where resistance determinants may be maintained even when recent antimicrobial exposure is not documented [66,68]. *tet*C was found exclusively in WB-U (25.0%), although the difference among populations was not statistically significant. Neither *tet*B nor *tet*D was detected in any animal. The occurrence of tetracycline resistance genes in WB without direct antimicrobial exposure has been reported previously and has been attributed to contact with domestic animals or contaminated environmental matrices [25,67,69]. Taken together, these exploratory findings suggest that the detection frequencies of selected resistance genes may vary across the ecological contexts represented by the three sampled populations including possible differences in anthropogenic exposure [25,27,67], without demonstrating independent effect of anthropogenic exposure or domestication. WB-U was the only population in which all three detected determinants occurred. Moreover, *blaTEM* detection frequency was significantly higher in WB-U than in WB-S, despite the partial overlap between their ileal microbiota profiles. The resistance gene findings should be interpreted within the limits of the targeted approach used. PCR screening of total ileal DNA assessed only the occurrence of eight selected β-lactam and tetracycline resistance determinants and did not characterize the complete gut resistome. Moreover, the detected genes cannot be assigned to specific bacterial taxa on the basis of the parallel *16S rRNA* gene analysis [70]. Finally, because only 12 animals were examined per population, the observed detection frequencies describe the examined sample and should not be interpreted as population-level prevalence estimates. More broadly, the observed patterns should be interpreted at the level of the three sampled populations. Because WB-U represented a single urban population and anthropogenic exposure was not quantified, these comparisons describe the sampled ecological contexts but do not establish general effects of urbanization, anthropogenic exposure, or domestication. These exploratory findings suggest that WB-U may warrant consideration in future ARG surveillance at the wildlife–livestock–human–environment interface and underlines the public-health relevance of monitoring these populations as urban areas continue to expand [25,32].

## 5. Conclusions

The findings reveal differences in the composition of the ileal bacterial community and in the presence of specific antimicrobial resistance genes among the three sampled Sicilian suid populations. The high frequency of the *blaTEM* gene in urban wild boars is consistent with a possible influence of environments affected by human activity; however, the presence of geographical and ecological factors, as well as the absence of direct exposure measurements, precludes the establishment of a causal relationship. Large-scale studies incorporating spatial replication, detailed individual-level metadata, environmental sampling, and broader resistome characterization are required to confirm these observations and evaluate the potential value of urban wild boars as sentinels for antimicrobial resistance.

## Figures and Tables

**Table 1:** Primer pairs used for the amplification of β-lactam and tetracycline resistance genes.

Target Gene	Primer Sequence (5′ → 3′)	Amplicon Size (bp)	Tm (°C)	Reference
***blaTEM***	AGTGCTGCCATAACCATGAGTG CTGACTCCCC GTCGTGTAGATA	431 bp	61 °C	[40]
***blaCTX-M IV***	GACAAAGAGAGTGCAACGGATG TCAGTGCGATCCAGACGAAA	501 bp	61 °C	[40]
***blaOXA***	ATTATCTACAGCAGCGCCAGTG TGCATCCACGTCTTTGGTG	296 bp	61 °C	[40]
***blaSHV***	GATGAACGCTTTCCCATGATG CGCTGTTATCGCTCATGGTAA	214 bp	61 °C	[40]
***tetA***	TTGGCATTCTGCATTCACTC GTATAGCTTGCCGGAAGTCG	494 bp	57 °C	[41]
***tetB***	CAGTGCTGTTGTGTCATTAA GCTTGGAATACTGAGTGTAA	571 bp	57 °C	[41]
***tetC***	CTTGAGAGCCTTCAACCCAG ATGGTCGTCATCTACCTGCC	418 bp	59 °C	[41]
***tetD***	GCTCGGTGGTATCTCTGCTC AGCAACAGAATCGGGAACAC	546 bp	59 °C	[41]

**Table 2:** Detection frequencies of selected ARGs in ileal samples from domestic pigs (DP), sylvatic wild boars (WB-S), and urban wild boars (WB-U) from Sicily. Values are reported as the number of animals in which the gene was detected/number examined (*n* = 12 per group), percentage, and exact 95% Clopper–Pearson confidence interval. Pairwise comparisons were performed using two-sided Fisher’s exact tests. All *p*-values were adjusted using the Holm–Šídák method. An asterisk indicates statistical significance at *p* < 0.05. A dash (—) indicates that the gene was screened but not detected.

Antimicrobial Class	Gene	DP, *n*/N (%; 95% CI)	WB-S, *n*/N (%; 95% CI)	WB-U, *n*/N (%; 95% CI)	Holm–Šídák *p*-Value Adjusted
**β-lactam**	*blaTEM*	6/12 (50.00%; 21.09–78.91%)	2/12 (16.67%; 2.09–48.41%)	10/12 (83.33%; 51.59–97.91%)	DP vs. WB-S: 0.3488; DP vs. WB-U: 0.3488; WB-S vs. WB-U: 0.0099 *
	*blaOXA*	—	—	—	
	*blaCTX-M IV*	—	—	—	
	*blaSHV*	—	—	—	
**Tetracycline**	*tetA*	5/12 (41.67%; 15.17–72.33%)	—	4/12 (33.33%; 9.92–65.11%)	DP vs. WB-S: 0.1078; DP vs. WB-U: 1.0000; WB-S vs. WB-U: 0.1777
	*tetB*	—	—	—	
	*tetC*	—	—	3/12 (25.00%; 5.49–57.19%)	DP vs. WB-S: 1.0000; DP vs. WB-U: 0.5207; WB-S vs. WB-U: 0.5207
	*tetD*	—	—	—	

## Data Availability

Data are available on NCBI under Bioproject accession number (PRJNA1505544).

## Supplementary Materials

The following supporting information can be downloaded at: https://www.mdpi.com/article/10.3390/ani16182938/s1, Table S1. Sample ID, environmental group, and geographical origin of the 36 suid samples included in the study. Table S2. Individual alpha diversity metrics of the gut bacterial communities. ACE, Chao1, Shannon, Simpson, Good’s coverage, and total ASV richness are reported for each domestic pig (DP), sylvatic wild boar (WB-S), and urban wild boar (WB-U) sample. Table S3. Detection of selected antimicrobial resistance genes in ileal samples from each domestic pig (DP), sylvatic wild boar (WB-S), and urban wild boar (WB-U). Results are reported separately for each animal. A plus sign (+) indicates gene detection, whereas a minus sign (−) indicates no detection.
