# Supplementary material for: Exploring Gut Microbiota Composition and Occurrence of Selected Antimicrobial Resistance Genes in Domestic Pigs and Urban and Sylvatic Wild Boars from Sicily
Source: Animals (Basel). 2026 Sep 18;16(18):2938. doi: 10.3390/ani16182938 (PMC13603938; doi:10.3390/ani16182938)
Supplement: Supplementary file 1 [file animals-16-02938-s001.zip › animals-4505213-supplementary.pdf]

animals

MDPI

Supplementary material

Exploring gut microbiota composition and occurrence of selected antimicrobial resistance genes in domestic pigs and urban and sylvatic wild boars from Sicily

Martina Maria Imburgia[1†], Fanny Claire Capri [2,3,†], Rosa Alduina [2,3,*], Roberto Puleio[1], Rosaria Disclafani[1], Valeria Blanda[1], Paola Galluzzo[1,*], Sergio Migliore[1,4]

[1] Istituto Zooprofilattico Sperimentale della Sicilia "Mirri", Via G. Marinuzzi 3, 90129 Palermo, Italy; mmartina.iimburgia@gmail.com (M.M.I.); roberto.puleio@izssicilia.it (R.P.); rosaria.disclafani@izssicilia.it (R.D.); valeria.blanda@izssicilia.it (V.B.); paola.galluzzo@izzsicilia.it (P.G.); sergio.migliore@izssicilia.it (S.M.)
[2] Dipartimento Scienze e Tecnologie Biologiche, Chimiche e Farmaceutiche, Viale delle Scienze, Università Di Palermo, 90128, Palermo, Italy; fannyclaire.capri@unipa.it (F.C.C.); valeria.alduina@unipa.it (R.A.)
[3] National Biodiversity Future Center (NBFC), Piazza Marina, 61, Palermo, 90133, Italy; fannyclaire.capri@unipa.it (F.C.C.); valeria.alduina@unipa.it (R.A.)
[4] Department of Veterinary Sciences, University of Messina, Viale Giovanni Palatuci 13, 98168 Messina, Italy; migliore.sergio@gmail.com (S.M.)

† these authors contributed equally to this work
* Correspondence: valeria.alduina@unipa.it; paola.galluzzo@izssicilia.it;

Table S1. Sample ID, environmental group, and geographical origin of the 36 suid samples included in the study

NO.	SAMPLES ID	ENVIRONMENTAL	GEOGRAPHICAL ORIGIN
**1**	1DP	Domestic	Caronia (ME)
**2**	2DP	Domestic	Caronia (ME)
**3**	3DP	Domestic	Caronia (ME)
**4**	4DP	Domestic	Caronia (ME)
**5**	5DP	Domestic	Caronia (ME)
**6**	6DP	Domestic	Nissoria (EN)
**7**	7DP	Domestic	Nissoria (EN)
**8**	8DP	Domestic	Caronia (ME)
**9**	9DP	Domestic	Caronia (ME)
**10**	10DP	Domestic	Caronia (ME)
**11**	11DP	Domestic	Caronia (ME)
**12**	12DP	Domestic	Sanfratello (ME)

**13**	1WB-U	Urban	Palermo
**14**	2WB-U	Urban	Palermo
**15**	3WB-U	Urban	Palermo
**16**	4WB-U	Urban	Palermo
**17**	5WB-U	Urban	Palermo
**18**	6WB-U	Urban	Palermo
**19**	7WB-U	Urban	Palermo
**20**	8WB-U	Urban	Palermo
**21**	9WB-U	Urban	Palermo
**22**	10WB-U	Urban	Palermo
**23**	11WB-U	Urban	Palermo
**24**	12WB-U	Urban	Palermo
**25**	1WB-S	Sylvatic	Collesano (PA)
**26**	2WB-S	Sylvatic	Castelbuono (PA)
**27**	3WB-S	Sylvatic	Castelbuono (PA)
**28**	4WB-S	Sylvatic	Collesano (PA)
**29**	5WB-S	Sylvatic	Castelbuono (PA)
**30**	6WB-S	Sylvatic	Collesano (PA)
**31**	7WB-S	Sylvatic	Collesano (PA)
**32**	8WB-S	Sylvatic	Collesano (PA)
**33**	9WB-S	Sylvatic	Collesano (PA)
**34**	10WB-S	Sylvatic	Collesano (PA)
**35**	11WB-S	Sylvatic	Collesano (PA)
**36**	12WB-S	Sylvatic	Castelbuono (PA)

Table S2. Individual alpha-diversity metrics of the gut bacterial communities. ACE, Chao1, Shannon, Simpson, Good's coverage, and total ASV richness are reported for each domestic pig (DP), sylvatic wild boar (WB-S), and urban wild boar (WB-U) sample.

Sample ID	Group	ace	chao1	shannon	simpson	goods_coverage	ASV
1DP	Domestic	52	52	3.18	0.76	1	52
2DP	Domestic	76	76	4.25	0.9	1	76
3DP	Domestic	18	18	2.82	0.77	1	18
4DP	Domestic	37	37	3.57	0.85	1	37
5DP	Domestic	37	37	3.68	0.86	1	37
6DP	Domestic	20	20	1.19	0.28	1	20
7DP	Domestic	76.8	76	3.55	0.75	0.99	76
8DP	Domestic	29	29	3.41	0.85	1	29
9DP	Domestic	20	20	2.46	0.65	1	20
10DP	Domestic	38.58	38	3.21	0.76	0.99	38
11DP	Domestic	38.6	38	3.37	0.8	0.99	38
12DP	Domestic	21	21	2.73	0.79	1	21
1WB-S	Sylvatic	19	19	1.57	0.37	1	19
2WB-S	Sylvatic	23	23	2.17	0.64	1	23
3WB-S	Sylvatic	16	16	1.98	0.54	1	16
4WB-S	Sylvatic	23	23	3.46	0.85	1	23
5WB-S	Sylvatic	42	42	4.4	0.92	0.99	42
6WB-S	Sylvatic	39.12	38	3.05	0.75	1	38
7WB-S	Sylvatic	18	18	2.76	0.75	1	18
8WB-S	Sylvatic	16	16	2.42	0.73	1	16
9WB-S	Sylvatic	17	17	1.49	0.41	1	17
10WB-S	Sylvatic	11	11	1.6	0.54	1	11
11WB-S	Sylvatic	28	28	2.95	0.8	1	28
12WB-S	Sylvatic	26	26	3.04	0.83	1	26
1WB-U	Urban	27	27	3.11	0.82	1	27
2WB-U	Urban	11	11	1.71	0.6	1	11
3WB-U	Urban	19	19	2.08	0.6	1	19
4WB-U	Urban	23	23	1.75	0.43	1	23
5WB-U	Urban	23	23	3.41	0.86	1	23
6WB-U	Urban	28	28	3.96	0.91	1	28
7WB-U	Urban	56	56	4.96	0.95	1	56
8WB-U	Urban	41	41	4.17	0.91	1	41
9WB-U	Urban	19	19	2.65	0.75	1	19
10WB-U	Urban	16	16	3.08	0.84	1	16
11WB-U	Urban	13	13	1.98	0.61	1	13
12WB-U	Urban	31	31	3.78	0.89	1	31

Table S3. Detection of selected antimicrobial resistance genes in ileal samples from each domestic pig (DP), sylvatic wild boar (WB-S), and urban wild boar (WB-U). Results are reported separately for each animal. A plus sign (+) indicates gene detection, whereas a minus sign (−) indicates no detection.

Sample ID	Group	*blaTEM*	*blaOXA*	*blaSHV*	*blaCTX-M IV*	*tetA*	*tetB*	*tetC*	*tetD*
**1 DP**	Domestic	-	-	-	-	-	-	-	-
**2 DP**	Domestic	-	-	-	-	-	-	-	-
**3 DP**	Domestic	+	-	-	-	-	-	-	-
**4 DP**	Domestic	-	-	-	-	-	-	-	-
**5 DP**	Domestic	+	-	-	-	-	-	-	-
**6 DP**	Domestic	+	-	-	-	-	-	-	-
**7 DP**	Domestic	+	-	-	-	-	-	-	-
**8 DP**	Domestic	-	-	-	-	+	-	-	-
**9 DP**	Domestic	+	-	-	-	+	-	-	-
**10 DP**	Domestic	+	-	-	-	+	-	-	-
**11 DP**	Domestic	-	-	-	-	+	-	-	-
**12 DP**	Domestic	-	-	-	-	+	-	-	-
**1 WB-S**	Sylvatic	+	-	-	-	-	-	-	-
**2 WB-S**	Sylvatic	+	-	-	-	-	-	-	-
**3 WB-S**	Sylvatic	+	-	-	-	-	-	-	-
**4 WB-S**	Sylvatic	-	-	-	-	-	-	-	-
**5 WB-S**	Sylvatic	-	-	-	-	+	-	+	-
**6 WB-S**	Sylvatic	+	-	-	-	+	-	+	-
**7 WB-S**	Sylvatic	+	-	-	-	-	-	-	-
**8 WB-S**	Sylvatic	+	-	-	-	-	-	+	-
**9 WB-S**	Sylvatic	+	-	-	-	+	-	-	-
**10 WB-S**	Sylvatic	+	-	-	-	-	-	-	-
**11 WB-S**	Sylvatic	+	-	-	-	-	-	-	-
**12 WB-S**	Sylvatic	+	-	-	-	+	-	-	-
**1 WB-U**	Urban	-	-	-	-	-	-	-	-
**2 WB-U**	Urban	-	-	-	-	-	-	-	-
**3 WB-U**	Urban	-	-	-	-	-	-	-	-
**4 WB-U**	Urban	+	-	-	-	-	-	-	-
**5 WB-U**	Urban	-	-	-	-	-	-	-	-
**6 WB-U**	Urban	-	-	-	-	-	-	-	-
**7 WB-U**	Urban	-	-	-	-	-	-	-	-
**8 WB-U**	Urban	-	-	-	-	-	-	-	-
**9 WB-U**	Urban	-	-	-	-	-	-	-	-
**10 WB-U**	Urban	+	-	-	-	-	-	-	-
**11 WB-U**	Urban	-	-	-	-	-	-	-	-
**12 WB-U**	Urban	-	-	-	-	-	-	-	-
